# Deoxynivalenol and *Alternaria* Toxin Exposure and Health Effects Assessment of Pregnant Shanghai Women

**DOI:** 10.3390/foods14050776

**Published:** 2025-02-25

**Authors:** Kailin Li, Baozhang Luo, Hua Cai, Renjie Qi, Zhenni Zhu, Yi He, Aibo Wu, Hong Liu

**Affiliations:** 1 Shanghai Municipal Center for Disease Control and Prevention, Shanghai 200336, China; kailinli2023@163.com (K.L.); luobaozhang@scdc.sh.cn (B.L.); caihua@scdc.sh.cn (H.C.); qirenjie@scdc.sh.cn (R.Q.); zhuzhenni@scdc.sh.cn (Z.Z.); heyi@scdc.sh.cn (Y.H.); 2 CAS Key Laboratory of Nutrition, Metabolism and Food Safety, Shanghai Institute of Nutrition and Health, University of Chinese Academy of Sciences, Chinese Academy of Sciences, Shanghai 200031, China

**Keywords:** deoxynivalenol, *Alternaria* toxins, health effects, pregnant women

## Abstract

Deoxynivalenol (DON) and *Alternaria* toxins (ATs) are two common types of mycotoxins in food. Although they are physiologically toxic to animals and various cell lines, data related to the exposure risks and health effects in the human population were still limited, especially for ATs. In this study, we combined food consumption data and human biomonitoring data of 200 pregnant volunteers from different districts of Shanghai to assess the exposure to DON and ATs. In addition, correlations between food consumption and urinary DON and ATs levels, urine biomarkers, and blood indexes were analyzed by regression analysis. For DON, the exposure assessment of the probable daily intake (PDI) indicated that a portion (37.5%) of all participants exceeded the Tolerable Daily Intake (TDI) proposed for DON. For ATs, the PDI values estimated based on the urinary concentrations indicated that 2–100% of all participants exceeded the threshold of toxicological concern (TTC) values for ATs. In addition, we innovatively found some associations between exposure to ATs and abnormal uric acid and high-density lipoprotein cholesterol indexes by regression analysis. Despite the inevitable uncertainties, these results make an important contribution to the understanding of DON and ATs exposure risks and potential health hazards in the pregnant women population.

## 1. Introduction

Deoxynivalenol (DON) and *Alternaria* toxins (ATs) are commonly found in various food categories, especially cereal grains and their by-products [1]. DON was usually considered an enteropathogenic compound due to its relationship to gastrointestinal diseases and immunotoxicity [2,3]. In addition to causing animal diseases, DON-contaminated food can also cause food poisoning in humans, with symptoms of nausea, diarrhea, and vomiting [4]. Regarding the hazardous health effects of DON, the European Food Safety Authority (EFSA) has set a group Tolerable Daily Intake (TDI) of 1 µg/kg·bw/day for the sum of DON and its acetylated and glycosylated derivatives [5].

Among ATs, alternariol (AOH), alternariol monomethyl ether (AME), altenuene (ALT), tenuazonic acid (TeA), and tentoxin (TEN) have been validated to have significant physiological toxicity [6,7], and studies have shown that ATs not only damage host plant tissues but also specifically cause diseases in host plants, in addition to cytotoxicity, embryotoxicity, and teratogenicity following ingestion or administration in animals, mutagenicity in microbial and mammalian cell lines, and tumorigenicity in rats [8]. The EFSA has regarded ATs as potential risk factors to human health [9] and proposed the theoretical threshold of toxicological concern (TTC) values of 1500 ng/kg·bw/day for TeA and TEN, and 2.5 ng/kg·bw/day for AOH and AME, respectively [10,11].

Many studies based on dietary DON contamination and consumption data in different countries have shown that dietary exposure to DON in cereals, as well as in infant- and young-child foods, poses a health risk to adults [12,13,14], teenagers [12,15], and infants [16,17,18]. For the emerging ATs, studies on exposure risks are limited. However, the risk of dietary exposure to populations based on dietary ATs, contamination, and consumption data in certain areas has been demonstrated. Specifically, the estimated dietary exposure to AME and AOH in wheat grains was in the range of 0.003–0.007 µg/kg bw/day, exceeding the TTC value of 0.0025 µg/kg bw/day, demonstrating potential dietary risks for Chinese consumers [19]. In addition, ATs contamination in infant food also poses health risks to infants. A study from China found that TeA, AOH, and AME were detected in 47.5%, 7.5%, and 5.7% of the cereal-based food samples for Chinese infants and young children. Moreover, 1.5% of individuals exceeded the corresponding reference value for TeA, and 24.1% and 33.5% exceeded the reference values for AME and AOH, respectively [17].

Human biomonitoring (HBM) is an effective approach to assess in vivo mycotoxin exposure, which monitors mycotoxin biomarkers in human biological samples (e.g., serum, urine, and breast milk) [20,21,22]. Based on current studies, DON is one of the highest risks of population exposure [23]. HBM data related to DON exposure are mainly reported in children and adolescents as well as adults, whereas it varies among human groups. It has been reported that children and adolescents had higher rates and levels of the estimated daily intake (EDI) to DON exceeding the TDI than adults [1,23,24,25,26]. Data of the Norwegian population showed that the mean EDI of DON was 590 and 730 ng/kg·bw/day for children, 210 and 310 ng/kg·bw/day for adolescents, and 180 and 170 ng/kg·bw/day for adults [24]. Data from Italy reported that the mean EDI of DON was 757 and 683 ng/kg·bw/day for children, 328 and 427 ng/kg·bw/day for adolescents, and 177 and 170 ng/kg·bw/day for adults. In addition, 7.5% of the Italian population exceeded the TDI, and 40% of children exceeded the TDI [25]. Data from the Portuguese population showed that the mean EDI of DON was 495 ng/kg·bw/day for children, 445 ng/kg·bw/day for adolescents, and 297 ng/kg·bw/day for adults, with 0.1% of the general population exceeding TDI for DON, 3.2% for children, and 6.0% for adolescents [1]. Data from China show that the mean EDI of DON was 2220 ng/kg·bw/day for 1–6-year-old children, 3800 ng/kg·bw/day for 7–12-year-old children, 2640 ng/kg·bw/day for adolescents, and 1520 ng/kg·bw/day for adults, with 55.81% of the total population exceeding TDI for DON [26].

Regarding pregnant women, studies related to DON exposure risks are scarcer than those of the general population, which may be due to the difficulty of the investigation caused by changes in physical status. However, the detection of DON in human bio-samples of pregnant women is common. In rural Bangladesh, 6.0% of the urine samples from pregnant women detected DON [27]. Also, 38.7% of the serum samples from 579 pregnant women in rural Ethiopia were found to be polluted by DON [28].

For ATs, there were only limited reports on exposure assessment by HBM. Urine samples from German adult individuals detected TeA [29,30], and Portuguese adults detected AOH [31]. In addition, ATs were detected in urine samples from Chinese populations [32]. An exposure and risk assessment study from Beijing reported that 96% of urine samples of adult objects had AME detected, and the calculated average daily intake value was five times the TTC value of AME [33]. Data from the Yangtze River Delta showed that 100.0%, 99.2–100.0%, 0.372%, and 1.12% of adult objects exceeded the TTC values for AOH, AME, TeA, and TEN, respectively, which revealed high potential health risks [9]. However, to our best knowledge, the risk of exposure to ATs in pregnant women is rarely reported.

Many studies have shown that mycotoxin exposure may cause negative health effects in pregnant women; higher levels of aflatoxin exposure were reported to be associated with lower rates of gestational weight gain, microcytic hypochromic anemia, neonatal jaundice, and low birth weight in neonates [34,35,36,37,38,39]. In addition, fumonisin (FB) exposure among pregnant women was reported to be correlated with neural tube defects in the offspring [40]. Although these limited conclusions require more evidence, they are sufficient to warn of the health threat of mycotoxins to the group.

Considering, as mentioned above, food consumption data were combined with human exposure data (DON, AOH, AME, ALT, TeA, and TEN) in urine samples from 200 pregnant women living in Shanghai, China, to characterize exposure risks and figure out the correlation between food consumption and urinary biomarkers. Specifically, the correlations between mycotoxin exposure and blood index were innovatively analyzed to infer the health hazards.

## 2. Materials and Methods

### 2.1. Participants

A subsample of 200 participants from the Shanghai Diet and Health Survey (SDHS) [41,42] collected between November 2016 and March 2017 was extracted and incorporated into mycotoxin human biomonitoring. The first-morning urines and fasting blood were collected following a standardized protocol. Ethical approval was obtained from the Shanghai Municipal Center for Disease Control and Prevention Ethical Review Committee (EC No. 2016–32, approval date: 18 November 2016). Informed consent of all participants in this study was obtained according to the Ethical Principles.

### 2.2. Food and Sociodemographic Questionnaires

Participants performed three consecutive 24 h (h) recalls. All foods consumed during the 3-day 24 h period were recorded and quantified. Food categories comprised “staple foods” (rice and related products, wheat and related products, maize and related products, potatoes, multigrain and related products, and fried pasta), “food and vegetables”, “dairy products”, “meats” (poultry and livestock), “bakery”, “eggs”, “soybean and related products”, “aquatic products”, and “nuts”.

Social-demographic data were collected in a format of closed questions, including age, marital status, completed years of education, professional situation, and household annual food expense.

### 2.3. Exposure Assessment of Total DONs and ATs

First-morning urinary total DON and AT biomarker levels were quantified using ultra-performance liquid chromatography with tandem mass spectrometry (UPLC–MS/MS) (TSQ VANTAGE, Thermo Fisher Scientific, Waltham, MA, USA), and the analytical method of DON was performed using the method reported by our previous study [43], and the analytical method of ATs was optimized based on previously described methods [9,32]. Details are shown in Supplementary Material Table S1. Mycotoxin concentration below the LOQ was replaced with half the LOQ for the subsequent statistical analyses.

The PDI was calculated according to the following equation:

PDI (ng/kg bw/day) = c × (V/bw) × (100/E),(1)

where

c = DON or AT concentration in the analyzed urine samples (ng/mL urine);
V = daily urine volume of 1.5 L/day;
bw = body weight (kg);
E = urinary excretion rate of mycotoxin in 24 h.

Regarding the excretion rate, DON was 70% [5], AOH was 8.3%, AME was 6.7%, TeA was 89%, and TEN was 0.9% [9]. Risk assessment was performed by comparing the PDI with the TDI for DON and TTC for ATs, respectively. Monte Carlo simulation was conducted to quantify the probability estimation distribution of DON, TEA, AOH, AME, and TEN using @Risk 7.0 (Palisade software, New York, NY, USA). The hazard coefficients (HQ) were evaluated by the ratio of probable daily intake (PDI) (based on the probability estimation) and TDI used to indicate the noncarcinogenic risk of exposure to each mycotoxin.

### 2.4. Modeling of Food Consumption and HMB Data

Correlation analysis was conducted by combining urinary HBM data of total DON, TEA, AOH, AME, ALT, and TEN (expressed as volume-weighted concentrations (ng/mL)) with food consumption obtained from food questionnaires. Firstly, both variables were compared as continuous variables using Spearman’s correlation coefficient analysis (*n* = 200). Food category variables associated with urinary total DON, TEA, AOH, AME, ALT, and TEN biomarkers (*p* < 0.2) were incorporated into multivariate analysis [1]. Then, log-transformed urinary biomarkers as dependent variables and the food consumption data as independent variables were considered for the Generalized Linear Model (GLM) analysis. If *p* < 0.1, variables were considered to contribute to the model.

### 2.5. Modeling of DON and AT HMB Data and Renal and Liver Function Index Data

Blood levels of “alanine transaminase (ALT)”, “aspartate aminotransferase (AST)”, “blood urea nitrogen (BUN)”, “creatinine (CRE)”, “uric acid (UA)”, “total cholesterol (TC)”, “total protein (TP)”, “thyroid globulin (TG)”, “high-density lipoprotein cholesterol (HDL-C)”, “low-density lipoprotein cholesterol (LDL-C)”, “C-reactive protein (CRP)”, “transferrin (TRF)”, “soluble transferrin receptor (sTfR)”, “ferritin (Fer)”, “folic acid (FA)”, and “albumin (ALB)” expressed as mole-weighted concentrations (nmol/L) were used for regression analysis. Urinary mycotoxin markers associated with blood index (*p* < 0.2) were incorporated into multivariate linear regression analysis. Then, log-transformed blood index values as dependent variables and the urinary mycotoxin markers as independent variables were considered for the GLM analysis. If *p* < 0.1, variables were considered to contribute significantly to the model.

For multiple logistic regression, the “higher-than-normal”, “normal”, and “lower-than-normal” states of routine hematuria indicators as dependent variables and the urinary mycotoxin markers as independent variables were considered for the ordinal logistic regression analysis. If *p* < 0.1, variables were considered to contribute significantly to the ordinal logistic regression model if *p* < 0.1.

Statistical analysis was performed with GraphPad Prism 8.0 (San Diego, CA, USA) and SPSS 24.0 (IBM, Chicago, IL, USA).

## 3. Results

### 3.1. Sociodemographic Characterization of Participants

Participants in our study (*n* = 200) were similarly distributed by the city districts. The details of the socio-demographic characteristics of the study participants are presented in Table 1. The mean age of participants was 29.45 years (SD = 4.15). A substantial majority of them were married (99.0%). Regarding the educational level, 69.0% had received tertiary school education. A large part of the participants (30.5%) were the professional and technical staff.

Regarding anthropometric measures, the mean weight of participants was 61.21 ± 9.82 kg, and the mean height of participants was 1.61 ± 0.05 m. In addition, 44.5% of the total sample population was overweight, and 11.0% were obese. The median values of systolic pressure and diastolic pressure were 111.0 (89.67–552.67) and 70.0 (53.33–116.00), respectively (Table 2).

### 3.2. DON and AT Urinary Biomarkers and Food Consumption Data

**Figure 1:**
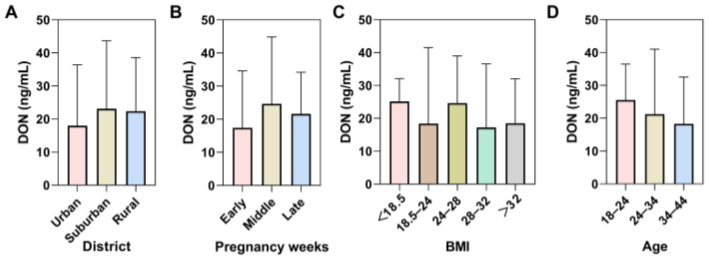
Concentrations of urinary total DON in different groups, according to (**A**) district, (**B**) pregnancy weeks; early pregnancy: 1–12 weeks; middle pregnancy: 13–27 weeks; late pregnancy: over 28 weeks, (**C**) BMI, and (**D**) age. Data were shown in the median with interquartile range. The *p*-value was calculated by the nonparametric test.

In urine samples, total DON was detected in all samples with a detection range of 0.57–358.35 ng/mL (mean: 33.91 ng/mL, median: 21.34 ng/mL). However, there was no statistically significant difference between the different districts, pregnancy weeks, BMI, and age groups (Figure 1).

**Figure 2:**
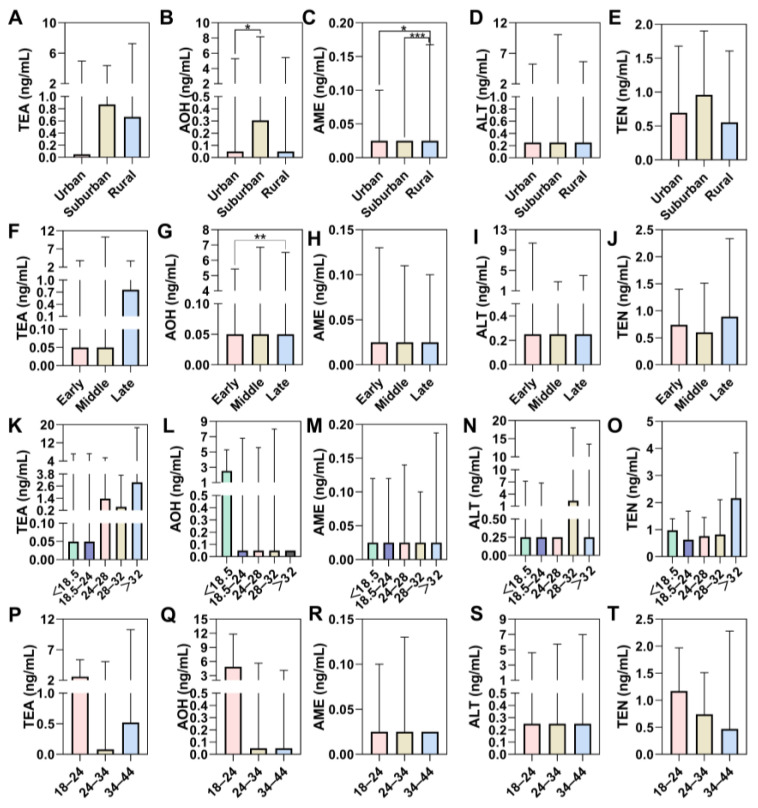
Concentrations of urinary ATs in different groups, according to (**A**–**E**) district, (**F**–**J**) pregnancy weeks; early pregnancy: 1–12 weeks; middle pregnancy: 13–27 weeks; late pregnancy: over 28 weeks, and (**K**–**O**) BMI, (**P**–**T**) age, figure (**A**,**F**,**K**,**P**) were for TEA, and (**B**,**G**,**L**,**Q**) for AOH, (**C**,**H**,**M**,**R**) for AME, (**D**,**I**,**N**,**S**) for ALT, and (**E**,**J**,**O**,**T**) for TEN. Data were shown in the median with interquartile range. *: *p*-value < 0.05; **: *p*-value < 0.01; ***: *p*-value < 0.001 by the nonparametric test.

Regarding ATs, positive samples (>limit of detection (LOD)) were detected for TEA (52.0%), AOH (43.0%), AME (34.0%), ALT (37.0%), and TEN (78.0%), and the detection ranges were ND–128.49 ng/mL (mean: 6.88 ng/mL, median: 0.36 ng/mL), ND–56.83 ng/mL (mean: 3.59 ng/mL, median: 0.05 ng/mL), ND–1.3 ng/mL (mean: 0.09 ng/mL, median: 0.025 ng/mL), ND–67.44 ng/mL (mean: 5.93 ng/mL, median: 0.25 ng/mL), and ND–30.31 ng/mL (mean: 1.36 ng/mL, median: 0.74 ng/mL), respectively. There was no statistically significant difference between different pregnancy weeks, BMI, and age groups. In Figure 2B, the median exposure level in the urine of AOH shows a significant difference between urban and suburban districts. In Figure 2C, the urine concentration of AME in rural districts is significantly different from the other two districts. In addition, AOH shows a significant difference between early and late pregnancy subjects (Figure 2G).

The food consumption data considered in the present study are reported in Supplementary Table S2. The highest consumption of food category was “vegetables and fruits”, followed by “staple food”, “dairy products”, “meats”, and “aquatic products”. In this study, we found that there was no significant difference in the consumption of these food categories between different pregnancy weeks except for the “dairy products” category, with women in late pregnancy having a higher intake of dairy products. In addition, the consumption of “bakery”, “livestock”, and “aquatic products” of pregnant women who lived in different regions showed statistically significant differences, subjects living in urban areas had higher livestock and aquatic products intake, regarding “bakery”, younger subjects had higher intake. However, there was no significant difference in the consumption of all categories of food except “soybean products” among pregnant women with different BMI ranges (Supplementary Table S2).

The results presented in Table 3 summarize the statistically significant correlations between the consumption of some food items and the urinary levels of DON and ATs (Table S3). Regarding the correlations, there is a predominance of negative correlation between food consumption and urinary mycotoxin levels. In addition, the significant correlations between TEA, AOH, and AME of ATs and “aquatic products” are, as far as we know, the first ever found (Table 3).

### 3.3. Exposure Levels of DONs and ATs

**Figure 3:**
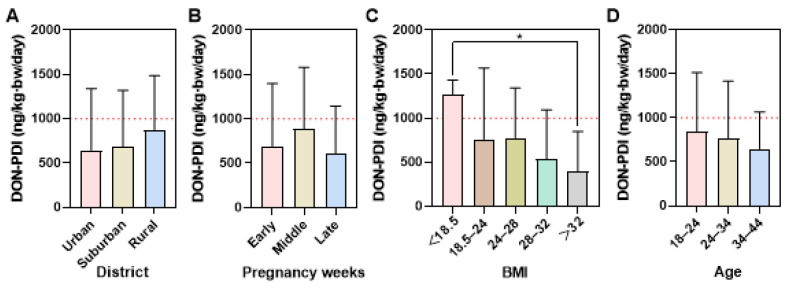
Comparison of PDI value of DON, according to (**A**) district, (**B**) pregnancy weeks; early pregnancy: 1–12 weeks; middle pregnancy: 13–27 weeks; late pregnancy: over 28 weeks, (**C**) BMI, and (**D**) age. Data were shown in the median with interquartile range. The *p*-value was calculated by the nonparametric test. Red dotted lines represent the TDI value of DON (1000 ng/kg·bw/day). *: *p*-value < 0.05 by the nonparametric test.

Using the DON and AT concentrations in urine samples (Table S3), the PDI values were calculated to assess the mycotoxin exposure. The median PDI value of subjects was 755.72 ng/kg·bw/day (mean: 1210.83 ng/kg·bw/day) with a range of 27.92–11,795.92 ng/kg·bw/day. A portion (37.5%) of the participants exceeded the TDI defined for DON. The distribution of PDI values of DON is shown in Figure 3. Although not statistically significant, rural subjects’ median PDI (876.50 ng/kg·bw/day) of DON was higher than in the other two districts; maternal mid-pregnancy PDI (888.10 ng/kg·bw/day) was higher than those at early (694.80 ng/kg·bw/day) and late (603.90 ng/kg·bw/day) pregnancy. In addition, younger subjects’ PDI was higher than in older groups (Figure 3A,B,D). However, underweight pregnant women’s median PDI value (1275.00 ng/kg·bw/day) was significantly higher than in obese groups (396.50 ng/kg·bw/day) (Figure 3C).

For ATs, the median PDI value of TeA was 11.02 ng/kg·bw/day (mean: 197.47 ng/kg·bw/day) with the range of 0.91–4021.56 ng/kg·bw/day, and the median PDI value of AOH was 17.63 ng/kg·bw/day (mean: 1115.44 ng/kg·bw/day) with the range of 9.04–19,942.68 ng/kg·bw/day, the median PDI value of AME was 10.53 ng/kg·bw/day (mean: 34.45 ng/kg·bw/day) with the range of 5.60–534.03 ng/kg·bw/day, and the median PDI value of TEN was 2002.69 ng/kg·bw/day (mean: 3675.70 ng/kg·bw/day) with the range of 10.62–73,746.96 ng/kg·bw/day. Notably, 2.0%, 100.0%, 100.0%, and 60.0% of the participants exceeded the TTC values for TeA, AOH, AME, and TEN, respectively.

**Figure 4:**
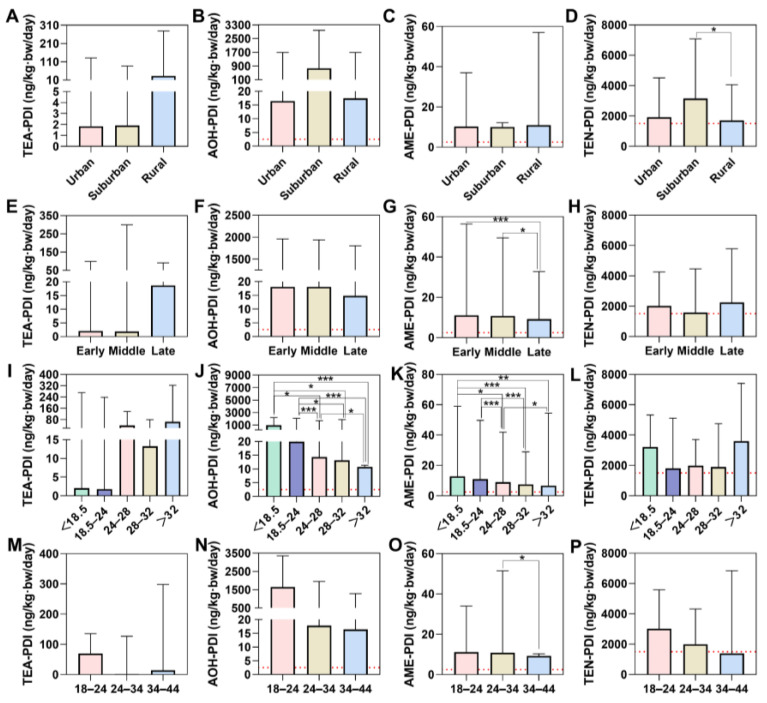
Comparison of PDI value of ATs, according to (**A**–**D**) district, (**E**–**H**) weeks of pregnancy; early pregnancy: 1–12 weeks; middle pregnancy: 13–27 weeks; late pregnancy: over 28 weeks, and (**I**–**L**) BMI, (**M**–**P**) age. Data were shown in the median with interquartile range. *: *p*-value < 0.05; **: *p*-value < 0.01; ***: *p*-value < 0.001; by the nonparametric test. Red dotted lines represent the TTC of TeA (1500 ng/kg·bw/day), AOH (2.5 ng/kg·bw/day), AME (2.5 ng/kg·bw/day), and TEN (1500 ng/kg·bw/day).

Statistically, there were no significant differences in the levels of TeA exposure among pregnant women in different groups. BMI may be the key factor in exposure levels of ATs, as there are significant differences in exposure levels of AOH and AME across different BMI ranges. The PDI of AME showed significant differences between different pregnancy and age groups. In addition, suburban subjects’ median PDI value of TEN (3146 ng/kg·bw/day) was higher than those in urban (1912 ng/kg·bw/day) and rural (1712 ng/kg·bw/day) areas. Similarly to DON, younger pregnant women’s median PDI values of ATs were higher than in other age groups (Figure 4).

### 3.4. Health Risk Assessment of DONs and ATs

**Figure 5:**
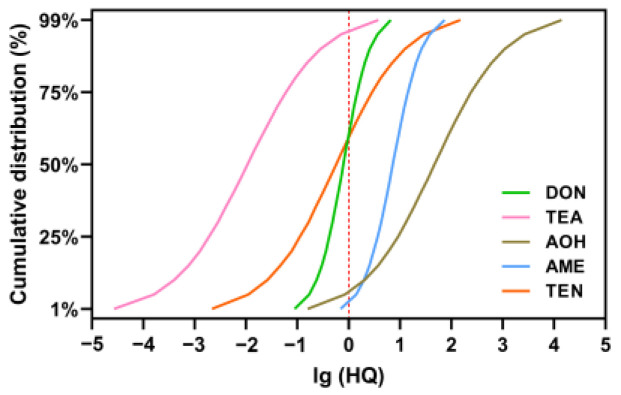
Monte Carlo simulation for DON and AT exposure to pregnant residents from Shanghai, China. Red dotted lines represent the PDI was equal to TDI for DON or the TTC for ATs (HQ = 1).

In our study, the HQ values were estimated to indicate the noncarcinogenic risks of DONs and ATs [44]. Among them, DON marginally exceeded the safe risk level (HQ = 1.21). For ATs, only TeA did not exceed safe risk levels (HQ = 0.29), but a small proportion of the population was exposed to health risks. AOH (HQ = 906.33), AME (HQ = 11.95), and TEN (HQ = 9.52) showed risks. In addition, the Monte Carlo simulations based on the simulated accumulation curve of lg (HQ) demonstrated that 39.6%, 95.0%, 97.6%, 3.8%, and 40.7% of the subjects had been, respectively, exposed to levels of DON, AOH, AME, TeA, and TEN, which constituted a health risk (lg (HQ) > 0) (Figure 5).

### 3.5. Link Between Mycotoxin Exposure and Renal and Liver Function Indices

To speculate on the health effects arising from DON and AT exposure, the correlations between urinary total DON and AT concentrations and blood index were analyzed in 200 subjects using GLMs. In Table 4, ATs presented a negative correlation with renal and liver function indexes. In addition, urine concentration of AME was negatively and linearly correlated with blood CRE, UA, and GLU (Table 4). The result indicates that the relationship between urinary AOH and blood FA content is also a negative linear relationship. Although there is a lack of information on toxicokinetic and excretion rates derived from humans for ALT, there were some negligible correlations between urinary ALT and blood index related to renal and liver function. Therefore, more attention needs to be devoted to the study of ALT.

Table 5 presents the model derived from multiple logistic regression using variables significantly associated with abnormal liver and kidney function indexes in the univariate analyses. In addition to the physical factors of the study subjects themselves, exposure to DONs and ATs may cause abnormalities in liver and kidney function indices in study subjects. AME exposure was a key factor in the lower-than-normal values of the TRF, UA, and VitB12 indicators. Urinary AME content was also confirmed to be linearly correlated with the values of UA through linear regression modeling (Table 4). ALT exposure was the main factor in the higher-than-normal values of the FA, whose presence increased the odds of above-normal FA by 1.755-fold (95% CI, 1.103–2.791). In addition, ALT not only correlates linearly with HDL-C but is also a key factor in the index being above normal values (OR, 0.524; 95% CI, 0.334–0.824). TEN was the main factor in the lower-than-normal values of ALB and higher-than-normal values of the TC and LDL-C indicators. Moreover, DON was also an important factor contributing to the abnormality of high LDL-C values.

## 4. Discussion

Mycotoxin exposure has received increasing attention in recent years, and while there are many studies assessing mycotoxin exposure in vitro through dietary exposure, there are few data on risk assessment through internal exposure, and most of them are currently focused on general populations such as children, adolescents, and adults, with a paucity of data on both mycotoxin exposure and health effects in the pregnant population. Accordingly, the present study was conducted to assess the risk of in vivo exposure as well as the health effects of commonly detected DON and ATs in food products in conjunction with dietary consumption data of pregnant women in Shanghai from the 2016–2017 SDHS and the results of biomonitoring of urine samples.

In the regression analysis of food consumption and urinary toxin concentrations of the 200 participants in this study, the results demonstrate a linear correlation between the consumption of aquatic products, poultry, eggs, and bakery items and the levels of urinary ATs. However, it is important to note that our evaluation of the daily exposure safety risk, which is based on urinary mycotoxin levels, is derived from the cumulative intake of all food items by the study population. Therefore, although the consumption of a specific food category is correlated with the concentration of urinary ATs, these associations may be influenced by carry-over effects or other uncertain factors and do not necessarily imply that the food category is hazardous. In fact, ATs are predominantly detected in grains (2–15,000 µg/kg) [45,46], tomatoes and related products (<0.26–41,389.19 µg/kg) [46,47,48], sunflower seeds and sunflower oil (mean value: 1.97–1618 µg/kg) [49,50], fruits and related products (1.32–54.89 µg/kg) [51,52], as well as beer (0.69–16.5 µg/L) [53] and wine [54]. Consequently, a higher intake of these ATs-contaminated food categories may pose potential risks.

For DON, the mean PDI was 1210.83 ng/kg·bw/day, which was higher than the PDI for adult populations in Europe and South America [1,23,24,25]. In addition, we found underweight pregnant women’s median PDI value was higher than other BMI range groups. For ATs, the median PDI value of TeA was 11.02 ng/kg·bw/day, AOH was 17.63 ng/kg·bw/day, AME was 10.53 ng/kg·bw/day, and TEN was 2002.69 ng/kg·bw/day; among them, 100% of the participants exceeded the TTC values for AOH and AME, which was comparable to data previously reported by China [9]. We applied HQ values to measure the noncarcinogenic risk of the two classes of toxins, and through Monte Carlo model construction analyses, we found that a significant proportion of the pregnant population in Shanghai is at health risk due to exposure to DONs and ATs. However, some uncertainties may have affected the risk assessment results. Firstly, due to the limited data for human excretion rates of ATs, exposure assessments were based on excretion rates in mice, which may result in an overestimation of the exposure risk. Secondly, the lack of TTC of ALT prevented the contaminant from being included in HQ calculations, and therefore the results of the risk assessment of ATs would be underestimated. In addition, assessing the health risks of AT exposure using the TTC as a reference value is more stringent and may yield a higher probability of risk than using the TDI. Thirdly, the calculated PDI represented the level of daily DON and AT exposure rather than appreciable risk over the entire pregnancy period. Hence, the non-carcinogenic health risks of DONs and ATs presented in our study may be overestimated, because of these uncertainties. Despite the uncertainties referred to above, these results provided significant risk assessment data on DON and AT exposure to the pregnant women population and consequently contributed to the prospective public health preventive measures.

Studies have indicated that exposure to mycotoxins may pose adverse health risks to pregnant women. This conclusion is drawn from analyzing the correlations between mycotoxin exposure and the physical conditions of pregnant women. For instance, there are associations between exposure to AFs and lower rates of gestational weight gain [34], anemia, and especially microcytic hypochromic anemia, across different trimesters of pregnancy [35]. Nonetheless, inferring the health hazards of mycotoxin exposure merely from physical condition outcomes like weight gain, vomiting, etc., may not be entirely accurate because the factors leading to changes in physical conditions during pregnancy are more intricate and multifaceted. In this study, we innovatively conducted an analysis of the association between mycotoxin markers in urine and indicators of liver and kidney function in blood collected at the same time period for each individual, which may be more convincing than associations between mycotoxin exposure and physical status or birth outcomes. Through linear regression analysis, we found that exposure to ATs in urine directly affected UA and HDL-C markers in liver and kidney function, and furthermore, the effect of ATs on abnormal UA and HDL-C markers was again verified in combination with logistic regression analysis. These findings could potentially shed light on the exploration of relevant mechanisms.

## 5. Conclusions

The pregnant population is an especially vulnerable group when it comes to dietary contamination exposure. In this study, we examined the exposure of pregnant women to DONs and ATs, two prevalent contaminants in foodstuffs, as well as the attendant health risks. Our findings indicated that the pregnant population in Shanghai was regularly exposed to DONs and ATs, with urinary exposure levels closely linked to food consumption patterns. Furthermore, a significant proportion of pregnant women in Shanghai were estimated to have exceeded the TDI/TTC, posing potential risks of liver and kidney function impairment. Accordingly, mycotoxin exposure in the population of pregnant women needs to be further explored. In summary, we highlighted the importance of conducting targeted assessments of mycotoxin exposure in highly sensitive populations. These assessments make it possible to identify the pregnant population and evaluate temporal trends in exposure. If necessary, the precautionary principle should be applied to implement control strategies for food contamination, thereby safeguarding the well-being of pregnant women and public health at large.

## Figures and Tables

**Table 1:** Socio-demographic characterization of participants.

Parameter	Number (*n* = 200)
Age in year	
Mean ± SD	29.54 ± 4.15
Median (Range)	29.00 (18.20–40.30)
18–24	16 (8.00)
24–34	153 (76.50)
34–44	31 (15.50)
Marital status	
Married	198 (99.00)
Never married	2 (1.00)
Occupation	
Enterprises and public institutions	9 (4.50)
Handle affairs personnel and concerned personnel	34 (17.00)
Professional and technical staff	61 (30.50)
Business and service	29 (14.50)
Agricultural, forestry, animal husbandry, fishery, and water conservancy production personnel	1 (0.50)
Production and transportation equipment operators	6 (3.00)
Housewife	20 (10.00)
Unemployed	21 (10.50)
Others	19 (9.50)
Education status	
Primary	1 (0.50)
Secondary	61 (30.50)
Tertiary	138 (69.00)

**Table 2:** Anthropometric characteristics of participants.

Parameter	Number
Weight in kilograms	
Mean ± SD	61.21 ± 9.82
Median	60.00 (41.70–100.00)
Height in meters	
Mean ± SD	1.61 ± 0.05
Median	1.60 (1.49–1.81)
BMI	
Mean ± SD	23.75 ± 3.55
Median	23.35 (17.11–35.62)
<18.5	11 (5.50)
18.5–24	100 (50.00)
24–28	67 (33.50)
28–32	17 (8.50)
>32	5 (2.50)
Systolic pressure	
Mean ± SD	113.68 ± 32.88
Median (range)	111.0 (89.67–552.67)
Diastolic pressure	
Mean ± SD	70.61 ± 8.24
Median (range)	70.0 (53.33–116.00)

**Table 3:** Correlations between consumption of food categories and the urinary levels of DONs and ATs.

Urine Biomarker	Food Category	Regression Coefficients	*p*-Value	R *
DON	Multigrain	−0.003	0.002	−0.213
DON	Nuts	−0.006	0.006	−0.300
TEA	Nuts	−0.012	0.083	−0.276
TEA	Aquatic products	0.003	0.069	0.208
AOH	Poultry	0.007	0.012	0.268
AOH	Aquatic products	−0.003	0.043	−0.305
AME	Aquatic products	−0.002	0.036	−0.312
ALT	Eggs	0.004	0.060	0.199
ALT	Bakery	0.002	0.082	0.193

* R = Spearman’s correlation coefficient.

**Table 4:** Multiple linear regression model to predict urine concentration of DONs and ATs and renal and liver function indices.

Index *	Mycotoxin	Regression Coefficients	*p*-Value	R *
CRE	AME	−0.039	0.003	−0.247
UA	AME	−0.031	0.105	−0.147
GLU	AME	−0.023	0.052	−0.155
FA	AOH	−0.031	0.030	−0.140
TG	ALT	−0.040	0.008	−0.174
HDL-C	ALT	−0.019	0.037	−0.203
CRP	ALT	−0.092	0.045	−0.150
TP	ALT	−0.009	0.082	−0.122

* CRE, creatinine; UA, transferrin; GLU, glucose; TG, thyroid globulin; HDL-C, high-density lipoprotein cholesterol; CRP, C-reactive protein; TP, total protein; FA, folic acid; R = Spearman’s correlation coefficient.

**Table 5:** Multiple logistic regression model for predicting the urine concentrations of DONs and ATs and abnormal liver and kidney function indices.

Index *	Mycotoxin	Regression Coefficient β (Standard Error)	OR * (95% CI *)	Wald Test	*p*-Value
TRF	AME	−1.093 (0.512)	0.335 (0.123–0.914)	4.561	0.033
UA	AME	0.083 (0.438)	2.292 (0.971–5.409)	3.586	0.058
VitB12	AME	1.370 (0.491)	3.934 (1.503–10.301)	7.779	0.005
ALB	TEN	0.515 (0.196)	1.673 (1.140–2.456)	6.920	0.009
TC	TEN	0.484 (0.243)	1.623 (1.007–2.614)	3.961	0.047
LDL-C	TEN	0.677 (0.355)	1.969 (0.982–3.948)	3.641	0.056
LDL-C	DON	1.222 (0.695)	3.395 (0.869–13.264)	3.092	0.079
FA	ALT	0.562 (0.237)	1.755 (1.103–2.791)	5.641	0.018
HDL-C	ALT	−0.646 (0.231)	0.524 (0.334–0.824)	7.822	0.005

* TRF, transferrin; UA, uric acid; ALB, albumin; TC, total cholesterol; LDL-C, low-density lipoprotein cholesterol; FA, folic acid; HDL-C, high-density lipoprotein cholesterol; OR, odds ratio; CI, confidence interval.

## Data Availability

The original contributions presented in the study are included in the article/Supplementary Material, further inquiries can be directed to the corresponding authors.

## Supplementary Materials

The following supporting information can be downloaded at https://www.mdpi.com/article/10.3390/foods14050776/s1, Table S1: Details of analytical method for determination of mycotoxins’ urinary biomarkers; Table S2: Mean food intake (g/day) of pregnant women; Table S3: Concentrations (ng/mL) of urinary DON and *Alternaria* toxins of 200 subjects in this study.

## Abbreviations

The following abbreviations are used in this manuscript:

DON	deoxynivalenol
ATs	*Alternaria* toxins
PDI	probable daily intake
TDI	tolerable daily intake
AOH	alternariol
AME	alternariol monomethyl ether
TeA	tenuazonic acid
ALT	altenuene
TEN	tentoxin
TTC	toxicological concern
EFSA	European Food Safety Authority
HBM	human biomonitoring
FBs	fumonisins
SDHS	Shanghai diet and health survey
UPLC–MS/MS	ultra performance liquid chromatography with tandem mass spectrometry
HQ	hazard coefficients
GLM	generalized linear model
ALT	alanine transaminase
AST	aspartate aminotransferase
BUN	blood urea nitrogen
CRE	creatinine
UA	uric acid
TC	total cholesterol
TP	total protein
TG	thyroid globulin
HDL-C	high-density lipoprotein cholesterol
LDL-C	low-density lipoprotein cholesterol
CRP	C-reactive protein
TRF	transferrin
sTfR	soluble transferrin receptor
Fer	ferritin
FA	folic acid
ALB	albumin
LOD	limit of detection
