# Supplementary material for: Deoxynivalenol and Alternaria Toxin Exposure and Health Effects Assessment of Pregnant Shanghai Women
Source: Foods. 2025 Feb 25;14(5):776. doi: 10.3390/foods14050776 (PMC11898465; doi:10.3390/foods14050776)
Supplement: Supplementary file 1 [file foods-14-00776-s001.zip › foods-3450011-supplementary.pdf]

Article

Deoxynivalenol and *Alternaria* toxins exposure and health effects assessment of pregnant Shanghai women

Kailin Li [1,2], Baozhang Luo [1], Hua Cai [1], Renjie Qi [1], Zhenni Zhu [1], Yi He [1], Aibo Wu [2,*] and Hong Liu [1,*]

[1] Shanghai Municipal Center for Disease Control and Prevention, Shanghai, 200336, China; kail-inli2023@163.com, luobaozhang@scdc.sh.cn, caihua@scdc.sh.cn, qirenjie@scdc.sh.cn, zhuzhenni@scdc.sh.cn, heyi@scdc.sh.cn, liuhong@scdc.sh.cn

[2] CAS Key Laboratory of Nutrition, Metabolism and Food Safety, Shanghai Institute of Nutrition and Health, University of Chinese Academy of Sciences, Chinese Academy of Sciences, Shanghai, 200031, China; kail-inli2023@163.com, abwu@sinh.ac.cn

* Correspondence: abwu@sinh.ac.cn; Tel.: +86-21-54920716, liuhong@scdc.sh.cn; Tel.: +86-21-62758710

Academic Editor: Firstname Lastname

Received: date
Revised: date
Accepted: date
Published: date

Citation: To be added by editorial staff during production.

Copyright: © 2025 by the authors. Submitted for possible open access publication under the terms and conditions of the Creative Commons Attribution (CC BY) license (https://creativecommons.org/licenses/by/4.0/).

Supplementary Material–S1–Details of analytical method for determination of urinary mycotoxin biomarkers

Chemicals and reagents

Mycotoxin standards deoxynivalenol (DON), isotope-labelled internal standards (^{13}C) deoxynivalenol (^{13}C-DON), tenuazonic acid (TEA), alternariol (AOH), alternariol monomethyl ether (AME), tentoxin (TEN), altenuene (ALT), isotope-labelled internal standards ($^{13}C10$) TEA, isotope-labelled internal standards ($^{13}C14$) AOH, isotope-labelled internal standards TEN-d6, and isotope-labelled internal standards ($^{13}C15$) ALT were purchased from Romer Labs (Union, MO, USA). Mycotoxin solid standards were dissolved in methanol or acetonitrile according to the respective certificate of analysis, and were stored at -18 °C. Enzyme β-glucuronidase/arylsulfatase (β-Gluc/ArylS) from *E. coli* was purchased from Sigma Aldrich (St. Louis, MO, USA).

Sample preparation

For ATs, urine samples were thawed at room temperature for 30 min and vortexed for 2 min. Then, 1 mL of each sample was digested with 20 μL β-Gluc/ArylS and 1 mL 0.2 mol/L sodium acetate buffer (pH 5.0–6.0) in a water bath at 37 ◦C overnight. The digested sample was adjusted to about pH 3 with HCl, and then mixed with 5 mL of ethyl acetate and vortexed for 2 min. After centrifugation at 5,000 rpm/min for 15 min, 4 mL of the upper organic phase was transferred into a new centrifuge tube. Then it was mixed with 5 mL of ethyl acetate and vortexed for 2 min again. After another centrifugation at 5,000 rpm/min for 15 min, 5 mL of the upper organic phase was transferred into a centrifuge tube and dried under a stream of nitrogen at 40 ◦C. The residues were re-dissolved in 200 μL of methanol and centrifugation at 12,000 rpm/min for 15 min and passed through PTFE membrane syringe filters (0.22 μm) before UPLC-MS/MS analysis.

Instrumental analysis

UPLC-MS/MS analysis was conducted on a a triple quadrupole mass spectrometer (TSQ VANTAGE, Thermo Scientific, USA).

For ATs, separation was achieved on an Agilent Extend C18 Column (4.6 mm × 150 mm, 3.5 μm) with the mobile phase consisting of water containing 2 mmol/L ammonium hydrogen carbonate (A) and methanol (B). A linear gradient elution program was designed as follows: initial 95% (A), 6 min 10% (A), 7 min 10% (A), 9 min 95% (A) and held for a further 3 min for re-equilibration, yielding a total run time of 12 min. The flow rate was 0.35 mL/min, and the injection volume was 10 μL. The column temperature and sample temperature were maintained at 40°C and 5 °C, respectively. DON were analyzed by MS/MS with the electrospray ionization source operated in positive (ESI+) mode. The parameters were set as follows: ion spray voltage, 3.5 kV (positive ion mode); source temperature, 300℃, sheath gas pressure, 30 psi, Auxiliary gas pressure: 20 psi; Capillary temperature: 350 ℃. Multiple reaction monitoring (MRM) acquisition mode was applied for the determination of the targeted analytes. The parameters and collision energies of precursor and product ions are listed in Table S1. MRM peak integrations and data analysis were carried out using the Xcalibur software (Thermo Fisher Scientific, 2011).

Table S1. MRM analysis parameters for mycotoxins.

Compound	Ionization mode	Precursor m/z	Product m/z	Collision voltage
DON	+	297.2	249.2	12
			231.2	15
^{13}C-DON	+	312.3	263.2	8
			245.1	10
TeA	-	196.1	138.84	21
			112.1	25
^{13}C10-TeA	-	206.1	144.8	20
			118.1	15
AOH	+	259.1	185.14	30
			128.13	49
$^{13}C_{14}$-AOH	+	273.1	197.15	30
			138.19	47
AME	-	271.1	228	29
			256	21
$^{13}C_{15}$-AME	-	286.1	270	23
			241	31
ALT	+	293.1	257.15	12
			115.11	50
$^{13}C_{15}$-ALT	+	305.78	289.95	20
			213.6	20
TEN	+	415.3	312.23	18
			256.23	25
TEN-D_6	+	421.2	315.2	21
			259.2	30

Table S2. Mean food intake (g/day) of pregnant women.

Food category	All subjects	Pregnancy weeks				Region			
		Early	Middle	Late	*p* value	Urban	Suburban	Rural	*p* value
Staple food									
Rice products	190.27 ± 103.95	176.8 ± 111.6	181.8 ± 81.72	214.5 ± 115.0	0.073	172.9 ± 84.82	232.0 ± 143.9	184.5 ± 91.03	0.121
Wheat flour products	82.82 ± 62.10	76.32 ± 51.55	88.33 ± 68.26	83.54 ± 65.38	0.834	86.52 ± 54.79	79.98 ± 62.65	81.34 ± 67.31	0.431
Maize products	5.62 ± 21.49	6.45 ± 23.98	5.15 ± 22.44	5.27 ± 17.48	0.787	4.09 ± 21.13	8.01 ± 21.97	5.68 ± 21.68	0.275
Potato	28.43 ± 44.72	26.74 ± 48.07	27.32 ± 35.80	31.53 ± 50.39	0.602	29.78 ± 43.12	32.27 ± 48.41	25.70 ± 44.50	0.505
Multigrain	17.80 ± 34.42	18.49 ± 35.16	20.32 ± 37.98	14.16 ± 29.17	0.765	18.34 ± 38.26	15.09 ± 27.26	18.61 ± 34.58	0.925
Fried pasta	3.40 ± 10.61	3.79 ± 11.33	3.59 ± 11.52	2.76 ± 8.68	0.940	3.89 ± 11.51	2.93 ± 9.34	3.25 ± 10.54	0.983
Bakery	37.35 ± 53.99	32.67 ± 40.19	31.58 ± 48.05	49.02 ± 70.08	0.309	29.87 ± 40.96	21.33 ± 38.93	50.16 ± 64.79	0.0052**
Soybean products	44.58 ± 60.24	43.18 ± 52.48	46.09 ± 70.55	44.34 ± 56.05	0.723	44.15 ± 62.74	32.60 ± 48.39	50.29 ± 62.88	0.293
Meats									
Poultry	25.42 ± 37.29	24.69 ± 28.06	24.77 ± 36.75	26.96 ± 46.17	0.663	24.19 ± 33.96	26.98 ± 44.47	25.64 ± 36.54	0.841
Livestock	85.70 ± 65.92	83.42 ± 65.08	85.04 ± 60.75	88.91 ± 73.09	0.915	106.3 ± 75.18	84.54 ± 60.13	70.86 ± 57.02	0.0036**
Eggs	47.35 ± 28.42	43.80 ± 25.76	47.13 ± 30.61	51.44 ± 28.50	0.275	54.39 ± 30.79	44.78 ± 22.31	43.25 ± 28.32	0.055
Dairy products	147.38 ± 129.77	119.7 ± 114.0	124.0 ± 106.7	204.0 ± 151.9	0.0004****	173.1 ± 150.0	139.9 ± 98.92	131.5 ± 123.9	0.188
Aquatic products	59.16 ± 64.88	65.68 ± 80.62	48.54 ± 46.91	64.27 ± 63.00	0.472	81.25 ± 77.55	55.34 ± 51.01	44.38 ± 55.47	0.0036**
Vegetables and fruits	446.74 ± 230.64	425.4 ± 214.7	428.9 ± 193.2	490.1 ± 279.4	0.443	479.7 ± 271.8	455.3 ± 202.9	418.2 ± 206.5	0.419
Nuts	14.64 ± 24.68	17.35 ± 24.90	13.31 ± 22.84	13.22 ± 26.57	0.217	13.42 ± 20.82	11.02 ± 20.39	17.17 ± 28.74	0.372

Table S2. Mean food intake (g/day) of pregnant women (continued).

Food category	BMI						Age			
	<18.5	18.5-24	24-28	28-32	>32	p value [a]	18-24	24-34	34-44	p value
Staple food										
Rice products	191.6 ± 106.3	177.9 ± 103.9	199.8 ± 78.47	229.4 ± 176.6	173.3 ± 59.35	0.326	184.5 ± 90.15	192.1 ± 95.55	184.3 ± 145.9	0.571
Wheat flour products	102.9 ± 79.86	78.54 ± 57.30	87.41 ± 65.15	77.04 ± 52.23	82.47 ± 107.8	0.726	81.02 ± 83.96	83.24 ± 61.55	81.68 ± 53.44	0.668
Maize products	16.97 ± 46.32	6.40 ± 22.89	3.19 ± 12.31	4.90 ± 20.21	0.00 ± 0.00	0.764	3.12 ± 9.07	6.94 ± 24.24	0.38 ± 2.10	0.238
Potato	38.18 ± 45.05	23.85 ± 41.74	26.79 ± 40.36	58.04 ± 68.97	20.00 ± 27.39	0.336	26.56 ± 32.09	32.53 ± 48.90	9.194 ± 12.90	0.130
Multigrain	26.52 ± 42.39	20.91 ± 38.62	12.51 ± 28.02	16.96 ± 27.73	10.00 ± 22.36	0.296	8.333 ± 16.19	18.55 ± 35.30	18.98 ± 36.91	0.664
Fried pasta	3.79 ± 10.11	2.69 ± 9.35	4.38 ± 12.49	2.53 ± 9.22	6.67 ± 14.91	0.816	3.42 ± 10.12	4.06 ± 11.60	0.16 ± 0.90	0.210
Bakery	52.42 ± 52.81	35.07 ± 49.78	33.72 ± 59.12	47.16 ± 58.80	65.33 ± 53.31	0.276	93.44 ± 104.5	34.46 ± 45.42	22.69 ± 38.46	0.0014**
Soybean products	56.06 ± 43.71	39.27 ± 58.38	56.53 ± 65.57	26.82 ± 58.87	25.67 ± 36.73	0.020*	49.06 ± 81.86	43.79 ± 60.48	46.16 ± 46.60	0.393
Meats										
Poultry	21.14 ± 16.06	26.60 ± 37.53	20.88 ± 32.24	43.92 ± 59.42	9.33 ± 14.61	0.184	15.00 ± 27.13	23.38 ± 32.99	40.86 ± 54.95	0.088
Livestock	122.8 ± 99.60	76.22 ± 54.97	94.27 ± 70.65	98.08 ± 75.33	36.67 ± 24.72	0.132	86.81 ± 62.88	85.61 ± 68.95	85.54 ± 52.49	0.673
Eggs	39.12 ± 27.92	44.67 ± 26.85	51.28 ± 28.89	48.03 ± 30.35	64.00 ± 44.56	0.504	32.71 ± 22.22	48.18 ± 28.09	50.83 ± 31.37	0.098
Dairy products	141.8 ± 90.18	125.1 ± 119.9	169.8 ± 149.7	175.7 ± 113.7	207.7 ± 106.4	0.084	147.1 ± 117.0	151.7 ± 131.9	126.5 ± 127.0	0.551
Aquatic products	34.09 ± 35.02	57.94 ± 70.53	56.83 ± 54.48	82.00 ± 79.24	92.33 ± 60.69	0.244	33.81 ± 29.32	59.00 ± 67.25	73.06 ± 63.49	0.126
Vegetables and fruits	548.9 ± 199.8	405.8 ± 185.1	453.9 ± 235.7	599.4 ± 369.7	425.1 ± 255.1	0.080	456.0 ± 239.3	456.0 ± 234.8	396.4 ± 204.3	0.495
Nuts	10.52 ± 13.92	13.72 ± 24.64	14.33 ± 25.52	15.47 ± 21.04	43.33 ± 34.32	0.176	14.27 ± 26.05	15.00 ± 25.56	13.01 ± 19.67	0.992

[a] p-values obtained using the Kruskal–Wallis test. $^{*}p < 0.05$, $^{**}p < 0.01$, $^{***}p < 0.001$, $^{****}p < 0.0001$.

Table S3. Concentrations (ng/mL) of urinary DON and *Alternaria* toxins of 200 subjects in this study.

Number	DON	TeA	AOH	AME	ALT	TEN
1	18.50	33.09	< LOD	< LOD	< LOD	2.16
2	10.90	< LOD	< LOD	0.12	< LOD	0.38
3	11.20	< LOD	< LOD	1.30	< LOD	1.00
4	93.30	2.90	< LOD	1.02	< LOD	3.03
5	37.50	9.63	< LOD	< LOD	< LOD	2.86
6	25.20	108.33	< LOD	0.12	< LOD	1.26
7	14.40	2.59	< LOD	0.17	< LOD	0.44
8	28.80	41.84	< LOD	< LOD	< LOD	1.05
9	68.60	89.25	< LOD	< LOD	< LOD	2.31
10	9.60	< LOD	< LOD	< LOD	< LOD	0.77
11	3.80	10.46	< LOD	0.14	< LOD	0.51
12	36.20	1.13	6.61	< LOD	< LOD	3.95
13	44.00	< LOD	6.85	0.27	4.55	1.27
14	8.80	< LOD	7.46	0.18	< LOD	0.16
15	31.70	2.83	13.59	< LOD	< LOD	1.38
16	24.30	< LOD	< LOD	< LOD	< LOD	1.32
17	8.90	4.38	< LOD	< LOD	< LOD	3.89
18	278.60	18.43	< LOD	< LOD	< LOD	1.00
19	27.60	< LOD	< LOD	0.18	< LOD	0.60
20	90.50	3.11	< LOD	< LOD	< LOD	3.15
21	42.40	< LOD	< LOD	< LOD	20.85	1.14
22	28.80	1.06	< LOD	< LOD	3.98	7.63
23	101.30	1.87	< LOD	< LOD	< LOD	2.87
24	7.40	< LOD	2.60	< LOD	4.56	2.47
25	3.40	< LOD	< LOD	< LOD	36.06	< LOD
26	3.20	0.35	11.15	< LOD	< LOD	2.31
27	17.00	0.36	13.52	< LOD	9.18	3.19
28	33.50	< LOD	< LOD	< LOD	17.83	1.80
29	18.50	1.38	< LOD	< LOD	< LOD	2.13
30	2.80	0.08	19.38	< LOD	< LOD	1.01
31	31.10	< LOD	19.84	0.12	< LOD	0.74
32	31.30	3.11	13.13	0.10	< LOD	30.31
33	10.00	< LOD	9.58	< LOD	12.55	3.06
34	17.20	< LOD	7.55	< LOD	18.16	3.14
35	12.60	< LOD	14.62	< LOD	39.59	0.87
36	13.20	< LOD	< LOD	0.10	12.35	3.23
37	17.40	4.37	< LOD	< LOD	19.72	0.75
38	9.30	< LOD	4.22	< LOD	10.37	0.63
39	51.00	2.81	1.52	< LOD	53.65	0.83
40	5.60	< LOD	0.56	< LOD	34.49	1.82
41	10.70	0.68	< LOD	< LOD	11.50	1.04
42	13.80	< LOD	13.79	< LOD	5.21	0.17
43	1.80	< LOD	19.29	< LOD	4.18	2.28
44	0.60	< LOD	4.37	< LOD	22.36	5.09
45	4.00	0.36	< LOD	< LOD	36.10	1.71
46	15.50	< LOD	3.74	< LOD	43.40	< LOD

Table S3. Concentrations (ng/mL) of urinary DON and *Alternaria* toxins of 200 subjects in this study (continued).

Number	DON	TeA	AOH	AME	ALT	TEN
47	114.50	10.56	5.97	< LOD	< LOD	2.50
48	10.70	< LOD	20.10	< LOD	14.78	< LOD
49	11.80	2.31	5.28	< LOD	< LOD	1.40
50	16.60	< LOD	8.41	0.10	11.32	0.49
51	123.50	6.61	< LOD	< LOD	34.40	7.28
52	21.90	< LOD	11.93	< LOD	27.75	0.85
53	18.30	3.99	< LOD	0.10	2.78	1.91
54	57.30	39.53	1.14	< LOD	39.92	6.46
55	16.10	< LOD	1.44	0.10	34.42	4.06
56	15.70	< LOD	7.93	0.10	14.72	2.97
57	20.70	1.00	< LOD	0.10	6.75	0.26
58	12.70	< LOD	< LOD	< LOD	25.94	0.51
59	6.90	0.34	< LOD	< LOD	< LOD	< LOD
60	34.10	< LOD	1.57	< LOD	4.89	1.69
61	29.50	< LOD	< LOD	< LOD	< LOD	< LOD
62	15.70	< LOD	2.26	< LOD	< LOD	< LOD
63	26.40	128.49	7.15	< LOD	32.62	< LOD
64	14.80	< LOD	4.61	< LOD	3.85	< LOD
65	30.30	< LOD	1.92	< LOD	25.63	< LOD
66	35.00	13.58	9.39	< LOD	< LOD	1.93
67	89.40	10.28	< LOD	< LOD	32.22	< LOD
68	46.00	< LOD	3.13	0.10	6.57	0.45
69	25.80	1.24	12.57	< LOD	1.70	< LOD
70	17.20	< LOD	< LOD	< LOD	< LOD	0.44
71	11.50	< LOD	10.51	< LOD	< LOD	< LOD
72	16.20	0.22	1.20	< LOD	5.10	1.05
73	15.30	< LOD	1.27	< LOD	< LOD	1.13
74	12.70	< LOD	< LOD	< LOD	< LOD	2.41
75	32.10	< LOD	2.53	< LOD	< LOD	0.16
76	10.40	< LOD	3.94	< LOD	< LOD	3.92
77	21.30	2.41	8.71	< LOD	< LOD	< LOD
78	25.30	< LOD	< LOD	< LOD	1.48	< LOD
79	38.90	15.95	< LOD	< LOD	9.95	< LOD
80	32.50	16.53	8.93	< LOD	15.04	4.96
81	7.00	< LOD	12.43	< LOD	< LOD	0.25
82	17.20	7.15	5.25	< LOD	5.96	0.53
83	3.50	< LOD	< LOD	< LOD	< LOD	0.67
84	32.30	2.84	9.57	< LOD	< LOD	< LOD
85	10.80	< LOD	9.57	0.10	1.42	< LOD
86	12.40	< LOD	7.60	0.10	11.47	0.96
87	71.60	< LOD	14.81	< LOD	< LOD	< LOD
88	13.10	0.55	0.90	< LOD	1.76	< LOD
89	11.60	< LOD	3.73	< LOD	1.11	< LOD
90	5.90	< LOD	< LOD	< LOD	< LOD	0.40
91	33.40	17.56	0.62	< LOD	< LOD	0.61
92	107.10	4.96	6.85	0.10	15.86	1.65

Table S3. Concentrations (ng/mL) of urinary DON and *Alternaria* toxins of 200 subjects in this study (continued).

Number	DON	TeA	AOH	AME	ALT	TEN
93	16.10	0.97	< LOD	0.10	< LOD	< LOD
94	16.30	< LOD	16.40	0.11	< LOD	< LOD
95	21.20	< LOD	5.43	< LOD	40.52	1.28
96	5.90	< LOD	< LOD	< LOD	5.50	0.32
97	11.90	4.15	< LOD	0.10	2.38	< LOD
98	6.70	< LOD	15.89	< LOD	6.65	1.07
99	32.30	3.78	< LOD	< LOD	< LOD	2.23
100	10.30	< LOD	8.70	< LOD	67.44	< LOD
101	8.60	< LOD	< LOD	< LOD	< LOD	0.09
102	28.10	< LOD	< LOD	< LOD	< LOD	2.09
103	13.80	2.37	4.35	< LOD	< LOD	0.57
104	39.90	4.92	< LOD	< LOD	< LOD	< LOD
105	149.10	67.34	< LOD	0.19	< LOD	1.40
106	18.30	4.32	< LOD	< LOD	< LOD	0.12
107	9.60	< LOD	< LOD	0.17	< LOD	1.51
108	12.30	3.80	< LOD	< LOD	5.29	0.15
109	18.00	< LOD	< LOD	0.37	4.16	0.88
110	12.40	< LOD	< LOD	< LOD	< LOD	1.40
111	21.80	< LOD	56.83	< LOD	< LOD	1.51
112	11.70	1.46	8.44	< LOD	< LOD	< LOD
113	11.30	< LOD	5.45	< LOD	< LOD	< LOD
114	39.00	27.37	5.62	< LOD	< LOD	0.47
115	11.60	< LOD	< LOD	< LOD	< LOD	0.63
116	16.20	3.40	< LOD	< LOD	< LOD	< LOD
117	81.10	47.62	< LOD	< LOD	< LOD	2.00
118	47.80	27.09	4.13	< LOD	6.43	0.16
119	32.40	35.69	< LOD	< LOD	< LOD	1.31
120	88.20	3.55	< LOD	< LOD	< LOD	0.26
121	26.60	3.25	< LOD	< LOD	3.34	0.90
122	11.90	12.33	< LOD	0.16	< LOD	1.41
123	45.4	2.51	< LOD	< LOD	< LOD	0.92
124	21.90	17.88	< LOD	< LOD	< LOD	0.20
125	44.90	< LOD	< LOD	0.13	< LOD	0.64
126	86.20	12.22	< LOD	0.34	< LOD	0.42
127	51.70	16.20	< LOD	< LOD	3.89	0.56
128	98.80	33.14	< LOD	< LOD	< LOD	1.35
129	16.70	18.18	< LOD	< LOD	< LOD	1.60
130	16.80	1.88	< LOD	< LOD	3.53	0.18
131	67.90	3.73	< LOD	< LOD	< LOD	1.14
132	127.20	17.43	< LOD	< LOD	< LOD	0.83
133	18.90	7.46	15.75	< LOD	< LOD	0.49
134	111.20	10.10	< LOD	0.13	< LOD	0.87
135	40.30	1.36	< LOD	< LOD	< LOD	< LOD
136	79.20	14.55	< LOD	< LOD	< LOD	0.76
137	48.90	< LOD	< LOD	< LOD	< LOD	< LOD
138	16.20	0.52	< LOD	< LOD	< LOD	< LOD

Table S3. Concentrations (ng/mL) of urinary DON and *Alternaria* toxins of 200 subjects in this study (continued).

Number	DON	TeA	AOH	AME	ALT	TEN
139	10.50	< LOD	5.67	0.21	< LOD	0.29
140	13.60	26.62	< LOD	< LOD	< LOD	< LOD
141	11.40	7.31	< LOD	< LOD	< LOD	0.29
142	28.70	19.17	4.58	0.14	< LOD	0.08
143	42.10	9.75	< LOD	0.21	< LOD	0.62
144	15.60	< LOD	14.52	0.17	< LOD	3.67
145	26.50	< LOD	< LOD	< LOD	< LOD	< LOD
146	114.50	< LOD	< LOD	0.27	< LOD	0.97
147	358.40	2.80	7.06	0.23	< LOD	1.63
148	49.80	2.55	< LOD	0.21	< LOD	< LOD
149	36.50	< LOD	< LOD	0.35	27.17	3.74
150	17.00	< LOD	< LOD	0.16	< LOD	0.11
151	41.60	2.98	< LOD	0.35	9.87	0.28
152	26.60	8.75	5.37	0.13	< LOD	0.26
153	34.60	2.54	8.10	0.30	< LOD	0.42
154	30.00	< LOD	< LOD	< LOD	0.69	< LOD
155	7.60	< LOD	0.57	< LOD	< LOD	< LOD
156	188.90	36.86	6.97	0.43	< LOD	1.05
157	40.40	1.70	4.74	0.13	< LOD	0.74
158	10.10	13.32	< LOD	0.14	6.98	< LOD
159	18.00	< LOD	6.48	0.16	< LOD	0.35
160	12.20	< LOD	< LOD	< LOD	< LOD	0.72
161	7.70	< LOD	< LOD	0.11	< LOD	< LOD
162	82.60	7.13	< LOD	0.51	1.93	2.97
163	22.40	< LOD	< LOD	< LOD	< LOD	3.8
164	25.00	0.48	11.25	0.14	< LOD	1.51
165	60.00	5.20	< LOD	0.17	14.07	4.29
166	42.8	7.16	< LOD	0.15	< LOD	< LOD
167	44.2	< LOD	< LOD	< LOD	< LOD	3.02
168	9.30	< LOD	3.64	0.16	< LOD	< LOD
169	50.70	3.21	< LOD	0.11	23.05	2.41
170	9.60	< LOD	< LOD	0.12	< LOD	0.11
171	21.30	32.3	9.00	< LOD	< LOD	9.96
172	11.80	3.24	< LOD	0.16	< LOD	5.30
173	53.60	< LOD	< LOD	< LOD	< LOD	1.65
174	24.80	< LOD	< LOD	0.11	7.18	0.07
175	23.80	< LOD	13.84	< LOD	< LOD	0.27
176	22.10	5.48	13.32	< LOD	< LOD	1.75
177	36.90	< LOD	< LOD	0.30	< LOD	0.54
178	34.30	1.23	< LOD	0.29	2.27	0.82
179	9.80	< LOD	8.10	< LOD	< LOD	< LOD
180	18.00	9.37	< LOD	0.12	< LOD	0.10
181	17.10	< LOD	8.53	0.18	< LOD	0.27
182	26.80	0.47	< LOD	< LOD	< LOD	1.74
183	127.80	17.77	< LOD	< LOD	< LOD	1.99
184	7.80	< LOD	< LOD	< LOD	< LOD	< LOD

Table S3. Concentrations (ng/mL) of urinary DON and *Alternaria* toxins of 200 subjects in this study (continued).

Number	DON	TeA	AOH	AME	ALT	TEN
185	37.20	12.10	5.29	< LOD	< LOD	0.75
186	16.80	< LOD	2.99	0.11	< LOD	< LOD
187	12.10	< LOD	< LOD	< LOD	17.14	1.21
188	26.50	10.84	< LOD	< LOD	< LOD	0.62
189	29.30	< LOD	5.38	0.14	< LOD	1.42
190	63.40	15.45	< LOD	0.34	< LOD	0.27
191	24.70	< LOD	5.58	< LOD	< LOD	1.45
192	45.40	45.90	< LOD	0.21	14.17	0.55
193	21.20	< LOD	7.65	0.56	20.81	0.51
194	6.40	< LOD	< LOD	< LOD	< LOD	< LOD
195	8.40	2.69	< LOD	< LOD	13.35	4.30
196	24.50	< LOD	< LOD	0.60	< LOD	3.14
197	41.90	< LOD	< LOD	0.35	20.68	0.66
198	16.10	< LOD	< LOD	< LOD	15.80	1.54
199	9.80	< LOD	< LOD	< LOD	< LOD	0.83
200	22.60	15.39	< LOD	< LOD	< LOD	0.19
